# In Vitro, In Vivo, and In Silico Investigation of Synbiotic-Mediated Activation of PPAR-*α* Curtails Nonalcoholic Steatohepatitis (NASH) in Wistar Rats by Inhibiting PNPLA3/SREBP1-c Lead Inflammatory Injury of Hepatic Cells

**DOI:** 10.1155/mi/9948679

**Published:** 2025-02-19

**Authors:** Dixa Sharma, Dhara Patel, Palash Mandal

**Affiliations:** Department of Biology, P. D. Patel Institute of Applied Sciences, Charotar University of Science and Technology, Changa, Anand 3888421, Gujarat, India

**Keywords:** inflammation, nonalcoholic steatohepatitis (NASH), oxidative stress, PNPLA3, PPAR-*α*, SREBP-1c, synbiotics

## Abstract

Nonalcoholic steatohepatitis (NASH) is an inflammation of the liver and a menace to human health. To treat NASH various pharmaceutical products have been used, but their prohibitive side effects limit their effectiveness. NASH, a multihit hypothesis involves high-fat diet and signals from the gut to the liver. *Lactobacillus plantarum* (probiotic) and aged garlic extract (AGE, a prebiotic) are antioxidative and anti-inflammatory and may be a latent combination therapy for NASH. The NASH model was developed using Wistar rats and treatments were administered to understand the mechanism. Initially, in the in vitro models, transepithelial electrical resistance (TEER) 2′-7′-dichlorodihydrofluorescein diacetate (DCFDA), 4-6-diamidino-2-phenylindole (DAPI) labeling and Oil Red O (ORO) conducted on HepG2 and Caco2 cells. Afterwards, in in vivo studies rat liver tissues were examined through confocal microscopy using the ORO staining and hematoxylin and eosin (H/E) stain, malondialdehyde (MDA), and biochemical indices were recorded. The levels of patatin-like phospholipase domain-containing protein 3 (PNPLA3) and sterol regulatory element binding protein-1c (SREBP-1c), peroxisome proliferators activated receptors (PPARs)-*α*, inflammatory, and apoptotic biomarkers were quantified by qRT-PCR. Synbiotic reduced the hepatic inflammation and apoptosis examined through the levels of PNPLA3, SREBP-1c, IL-6, TGF-*β*, Bcl-2, and caspase-3 in NASH models. In turn, the gram-negative species and bacterial translocation associated were reduced. Consequently, the Insilco analysis supports the theory that each (eight) bioactive compound of AGE targets PNPLA3 and enhances the PPAR-*α* activity. Additionally, PPAR-*α* inhibitors upregulated the PNPLA3 and SREBP-1C expression. As a result, the synbiotic may inhibit NASH progression by affecting PNPLA3/SREBP1-c through PPAR-*α*.

## 1. Introduction

Nonalcoholic steatohepatitis (NASH), a liver disease, occurs as a result of chronic oxidative and inflammatory stress. Several serious complications are associated with NASH, including hepatic cirrhosis [1, 2], hepatic failure [3], and hepatocellular carcinoma (HCC) [4, 5]. The clinical adverse effects related to NASH are regeneration of liver inhibition, infection, and others. To prevent and treat NASH and its complications, novel therapeutic methods are essential. There is no drug approved by the US Food and Drug Administration (FDA) to treat NASH without any negative effects [6]. NASH progression can still be controlled primarily with drug therapy. The commonly used drugs to treat NASH are peroxisome proliferators activated receptor (PPAR)-*α* and *γ* agonist [7], pioglitazone, [8] and vitamins [9], but these have many side effects.

A substantial amount of research has been conducted on the liver–gut axis in relation to NASH onset and progression [10]. Scientists may be able to mitigate the negative effects of this disease on the liver and general health by addressing gut health. During the metabolism process, the liver and intestinal environment are intimately connected via the portal circulation and packed two-way communication. The development of NASH is associated with increased intestinal permeability resulting from inflammation of intestinal tight junctions (TJs) [11]. TJs including occluding, claudin, and the occluding zone (ZO) regulate the permeability of intestinal mucosa [12]. There is an increase in intestinal permeability due to NASH which causes movement into the portal vein of bacterial endotoxins and contributes to liver inflammation [13–17]. As a result of de novo fatty acid synthesis, excess oleic acid (OA) also accumulates in the liver, causing hepatic steatosis. Whereas, the PPAR-*α* activation ameliorates NASH in hepatic and intestinal tissue through gene expression regulation.

Synbiotic, the combination of prebiotics and probiotics have significant anti-inflammatory and antioxidative properties. The garlic extract is rich in eight bioactive compounds, and thus, rich in various mineral ions and vitamins [18–21]. Anthocyanin in aged garlic extract (AGE) protects cells from inflammatory and diabetes-induced injuries through patatin-like phospholipase domain-containing protein 3/sterol regulatory element binding protein-1c (PNPLA3/SREBP-1c) pathway via modulating the PPAR-*α* levels [22]. The other phenolic compounds modulate the NF-*κ*B, TGF-*β*, TNF-*α*, IL-1*β*, IL-6, and IL-32. The AGE can be used as a prebiotic and in turn, affects the intestinal microflora. *Lactobacillus plantarum*, a probiotic, improves the intestinal microflora distribution [23].

Furthermore, inflammation and lipid metabolism may make fibrosis, atherosclerosis, and gut dysbiosis worse [23, 24], which in turn might facilitate the development of NASH [25]. The high levels of PNPLA3 and SREBP-1c lead to the NASH risk. Similarly, low levels of PPAR-*α* also affect the NASH progression, which was treated by seed extract of chicory through modulating the PPAR-*α* level [26], where activation of PPAR-*α* inhibited the SREBP-1c pathway [27]. PNPLA3 overexpression has been found in gut and liver tissue such as epithelial cells, enterocytes villi, paneth cells, and hepatocytes and has higher expression in insulin-resistant animal models [28].

Keeping the above described concept in vision, the present study examines oxidative stress, colonic barrier activity, fat accumulation, and inflammation associated with NASH progression in epithelial cells and Wistar rat models. The synbiotic used is unique and few data or no data on this combination is analyzed or reported on the NASH rat model. Results obtained in the current study suggest synbiotic may function as a therapeutic agent for NASH through PNPLA3 lead SREBP1-c pathway, investigated molecularly [29].

## 2. Materials and Methods

### 2.1. Reagents and Chemicals

AGE (Kyolic, Mission Viejo, CA, USA) and *L. plantarum* (MTCC 2621, MTCC India) have been used. Dulbecco's modified eagle medium (DMEM) has been fetched from Gibco, CA, USA; fetal bovine serum (FBS), OA, Primer sequences, and GW6471, an inhibitor of PPAR-*α*, were procured from Sigma–Aldrich, CA, USA. A synthesis kit for cDNA has been used and supplied by Applied Bio systems, CA, USA. Other chemicals were purchased through HiMedia and others from Mumbai (Maharashtra, India).

### 2.2. Cell Lines Culturing

*HepG2 and Caco2:* Both the cell lines were purchased from the National Centre for Cell Sciences (NCCS), India. Following the protocol described both the cell lines were cultured in DMEM [30] supplemented with 10% and 20% FBS according to the ATCC guidelines added with 100 µg/mL streptomycin under 5% CO_2_ at 37°C temperature.

### 2.3. HepG2 and Caco2 Experimental Dosing: NASH Model

To prepare the synbiotic combination *L*. *plantarum* (10^9^ CFU/mL) was cultivated at 37°C in a De Man, Rogosa, and Sharpe (MRS) medium. Nuclease-free water was used to suspend the cytosolic cell fraction (10^9^ CFU/mL) from the cell pellet obtained earlier. To generate the NASH model, cells were cultured with 25 mM glucose and 5 mM in DMEM along with 10^9^ CFU/mL of *L. plantarum* cell-free supernatant (MTCC 2621, MTCC India), 10 µg/mL working concentration of AGE, and 2 mM OA and 25 µg/mL of GW6471 were utilized as per the requirement by the groups for 24 h (Figure S1). The cell number was measured and adjusted with the help of a hemocytometer to 1 × 10^5^/mL and 180 µL was utilized in each well and kept for 48 h to culture before total RNA isolation [31, 32]. Trypsin (0.1%) and EDTA (0.4%) were used to detach the adherent cells after removing cell debris with nylon filters of 100 µm diameter. Each experiment was conducted using passages 21–29 of the cell line. The assigned groups are the control group (Control), NASH group (NM), PPAR-*α* antagonist (PA), synbiotic model (SM), and PPAR-*α* antagonist + synbiotic (PASM). Except control group, all were provided with NASH inducing diet as a base.

### 2.4. Caco2 Transepithelial Electrical Resistance (TEER) Analysis: NASH Model

To evaluate the barrier integrity of intestinal Caco2, cells were seeded in a transwell at 76,000–80,000 cells/cm^2^. For TEER assessment, in the upper and lower chambers, 0.5 and 1.5 mL of samples were applied according to the assigned groups. After the TEER was measured with helical volt ohmmeters [33]. To compare the mean resistance of treated and control cells devoid of inserts, cells devoid of inserts were designated as blank with no mean resistance.

### 2.5. In Vitro ROS Estimation and Lipid Profile Testing

An assay kit for detecting ROS generation in cells (ab113851, Abcam, CA, USA) was acquired to detect ROS generation with DCFD/H2DCFDA treated cells (24 h). Multimode plate readers analyzed the fluorescence intensities [34].

Both types of treated cells then fixed with 10% formalin reagent (1 h) to study lipid profiles. This methodology was mentioned by Patel et al. [19] in 2023. Afterward, to extract the stain, isopropanol (1 mL) was used. Absorbance was recorded for stain recovered at 570 nm under UV Spectrophotometer.

### 2.6. In Vitro Nuclear Condensation Study

Pretested or control cells were incubated in disease conditions for 24 h. The single-cell suspension of treated cells was washed and fixed with phosphate-buffered saline (PBS) and ethanol (70%), respectively. Cells were again flushed in PBS. After wash, cells were incubated with a 4-6-diamidino-2-phenylindole (DAPI) stain of 0.6 µg/mL in PBS for 5 min (ab228549, ABcam, USA). The fluorescence of chromatin was observed under a fluorescence microscope (Leica DMRB 2000). Morphological changes in apoptotic cells such as cytoplasmic and nuclear shrinkage and chromatin condensation were observed [35].

### 2.7. In Vivo Housing of Wistar Rats

The protocol and study were approved by the Animal Ethical Committee of Charotar University of Science and Technology (RPCP/IAEC/2023-24/R15). A total of 30 adult Wistar rats (200 ± 20 g) were used in the present study. Rats were housed (12-h day/night cycle, 55% ± 10% humidity, and 23 ± 2°C temperature). 22.5± mL/day/rat of water consumed by rats.

According to standard protocol, the animals were given chow diets and tap water (ad libitum) during the acclimatization period.

### 2.8. Methionine and Choline-Deficient High-Fat Diet (MCDHFD)-Induced NASH Model Development

The rats were fed with an MCDHFD (Table S3). In a 9-week study, the MCDHFD diet caused damage to the epithelial and intestinal barriers in the liver and gut and led to fat droplet accumulation in the liver. The rat NASH model was then assessed by a scoring system of histological features that scored the complete lesion spectrum of NASH [36, 37]. The synbiotics (*L. plantarum* 10^9^ CFU/day and 180 mg/kg of AGE) were administered orally to the required group, respectively [19]. While PPAR-*α* inhibitor (2 mM OA and 25 µg/mL of GW6471) was injected intraperitoneal into 12 rats. Following 40–60 mg/kg of pentobarbital as anesthetic compound, all fasting (12 h) rats underwent peritoneal blood (1–2 mL) collection before sacrifice from vena cava. Plasma was collected following centrifugation in Minispin-plus-Eppendorf, Hamburg, Germany, for 10 min at 16,000 relative centrifugal force (RCF), and the samples were then stored at −80°C for later use. The tissue samples of 20–30 mg were homogenized using a tissue homogenizer of Remi (RPM 8000 RQ-127AD) in PBS (150 μl) of pH 7.4.

**Figure 1:**
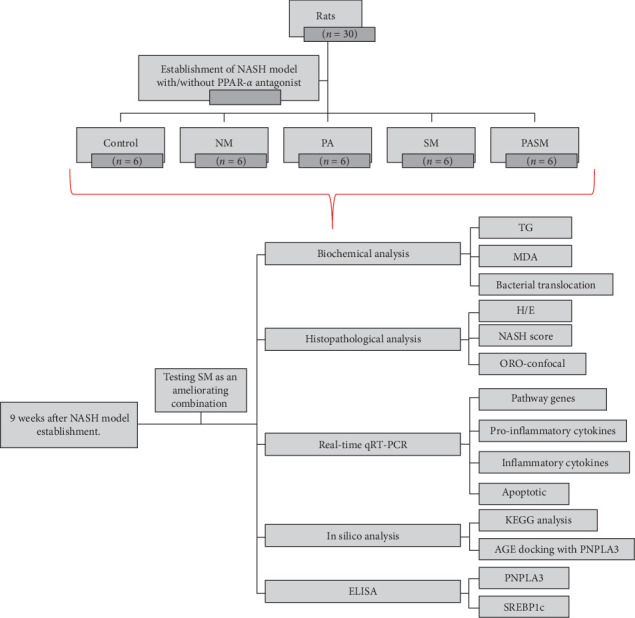
Study flow chart.

The animals were euthanized according to the animal ethical sacrifice protocol [19]. There were five experimental groups (six rats/group): the control group (Control), NASH group (NM), PPAR-*α* antagonist (PA), synbiotic model (NM + SM), and PPAR-*α* antagonist + synbiotic (PASM). Except control group all were provided with NASH inducing diet as a base (Figure 1).

### 2.9. Oil Red O (ORO) Stained Confocal Imaging of Lipogenesis

ORO was used to stain colon tissue samples mounted on glass slides. Confocal microscope (Leica laser, TCSSPI8) was then used for sample imaging. The ORO dye was imaged at 561 nm with laser 0.3% on a UV–visible spectrophotometer. The emission was collected from the dye by using a HyD detector at 80% gain at 572–669 nm. Confocal microscopic images were captured at 100 Hz (10,224 × 1024 format).

### 2.10. Liver and Colon Tissue Staining: Hematoxylin and Eosin (H/E) Staining

For morphological differentiation, H/E staining is performed on 5–10 µm of liver and colon sections. Sections were fixed in formalin and underwent optimal cutting temperature (OCT) treatment. Each slide section was then examined by researchers.

### 2.11. Real-Time Quantitative PCR Analysis

The Trizol method (Invitrogen, USA) was used to extract RNA by using a cDNA synthesis kit (BioradiScript). The obtained cDNA was reverse transcribed. The expression level of different markers such as PNPLA3, PPAR-*α* and SREBP1-c (deregulatory) Bcl2, Cas-3 (apoptotic), and IL-6 and TNF-*α* (inflammatory) mRNA was analyzed using a qRT-PCR Mx3005P machine (Agilent strata-gene, Hyderabad, India) and to generate gene expression SYBR/ROX was used (Primer's detail in Tables S1 and S2). NanoDrop (Thermo-Fisher Scientific, Waltham, USA), a spectrophotometer instrument was used to determine RNA concentration (ng/μL) and purity (A260/280). For endogenous control, the 18S rRNA gene was used. A fold-over analysis of basal level was performed comparing the results to the control group [31].

### 2.12. Bacterial Translocation and Intestinal Permeability Assessment

The MCDHFD and genetic factor-induced bacterial translocation were determined by mesenteric lymph nodes (MLNs) culture turbidity evaluation. The MLNs were aseptically removed from the rat's abdomen. Equal amounts of material were suspended in lysogeny broth (2 mL; #1278002, LB, Thermo Fisher Scientific, USA) that had been sterilized at 37°C for 6 h with gentle agitation. According to prior research, the difference in bacterial growth at 600 nm was evaluated spectrophotometrically [19].

### 2.13. Hepatic and Fecal Triglyceride Content Assessment

According to Folch's method, hepatic and fecal triglyceride extraction was performed. Using kits readily accessible on the market (#ab65336, Abcam, Cambridge, UK and ab178780, CA, USA), the absorbance has been measured with a spectrophotometer set to 570 nm [38].

### 2.14. Enzyme-Linked Immunosorbent Assay (ELISA)

Rat serum PNPLA3 and SREBP-1c levels were measured using an EIA-ADPN-1 ELISA kit (RayBiotech, CA, USA; cat. no. 38221990) and Abcam's SREBP-1 Transcription Factor Assay (Cambridge, UK; cat. no. ab133125), respectively. The intra- and inter-assay precision was less than 1.5%. The samples were treated and the manufacturer's guidelines were followed for analyzing the supernatant.

### 2.15. Pathway and Function Enrichment Analysis

Pathway and functional enrichment analysis were carried out using the Database for Annotation, Visualization, and Integrated Discovery (DAVID) VERSION 6.8 (http://david.ncifcrf.go) [36]. DAVID can offer the thorough and organized functional annotation tool required to uncover the biological significance of genes. The chosen downregulated genes, associated pathways, and upregulated genes by DAVID were examined using the Kyoto Encyclopedia of Genes and Genomes (KEGG) pathway enrichment analysis. *p*  < 0.05 is regarded as the statistical significance threshold.

### 2.16. MD of Pathway Protein PNPLA3

The 3D structure of the PNPLA3 10xw (Q9NST1.PLPL3_HUMAN) was found in the Protein Data Bank (uniport). SWISS model was used to construct the PNPLA3 homology model. Submitted protein structures were saved as pdbqt files. All ligands were found using PubChem. Optimum ligand molecules were determined by determining the torsion tree and number of rotatable bonds. Further docking studies were conducted using ligand data in pdbqt format generated earlier. For molecular docking simulations, lattices are crucial for guiding ligands to their target's proteins and active sites. In the default configuration, the lattice spacing was set to 0.375 inches. 41.856, 3.634, and 83.677 were the values for the mean lattice boxes. Grid points were set at 40 in each *x*, *y*, and *z* dimensions. Docking of the ligands with PNPLA3 was performed using AutoDock software 4.2.6 [67]. The model was visualized by using the software PyMOL.

### 2.17. Statistical Analysis

ANOVA (one-way) was used to evaluate the statistical data, followed by multiple comparison tests using Tukey post hoc tests and whisker plots to visualize the comparisons. A graph pad prism program (version 7.0, San Diego, USA) was used to graph the data. *p* values less than 0.05 are considered significant differences between groups.

## 3. Results

### 3.1. In Vitro

#### 3.1.1. Effect of and/or Synbiotic Intervention on the Growth of HepG2 Cells

**Figure 2:**
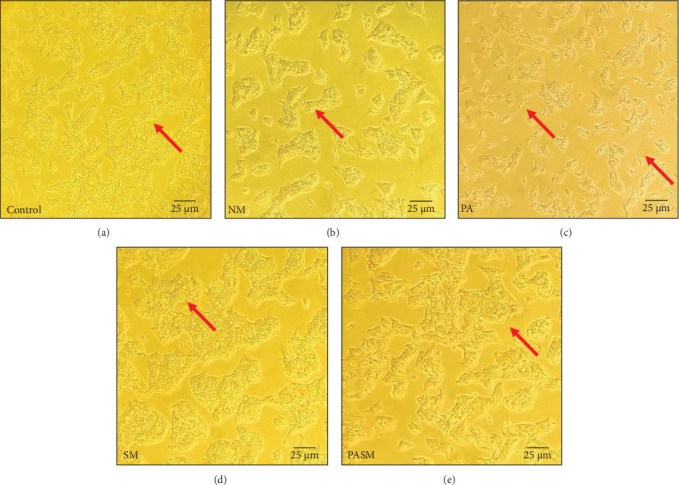
HepG2 cell morphology in each treatment group after 24 h. (A) Control group, (B) NM, (C) peroxisome proliferators activated receptor (PPAR)-*α* antagonist in HepG2 cells, (D) treatment with SM, and (E) PPAR-*α* antagonist cotreatment with SM. Arrows indicating apoptotic cells and changes in the characteristic epithelial morphology. The obtained results are representative of microscopic images of each group under the microscope (40x objective) with picture scale bar of 4.0 µm.

Hepatocellular uptake of plasma fatty acids and de novo biosynthesis in the liver contribute to liver fatty acid accumulation as proven already. To understand the basis of NASH progression due to MCDHFD, HepG2 cells were considered. A morphological examination of HepG2 cells was conducted after MCDHFD and PPAR-*α* antagonist treatment. The untreated HepG2 cells show their characteristic epithelial morphology (Figure 2A), while the PPAR-*α* antagonist-treated cells (Figure 2C) show constricted cell membranes and distorted cell shape. The untreated HepG2 cells show its characteristic epithelial morphology, whereas cells of the disease model (Figure 2B) show the affected growth rate of HepG2 cells after exposure to PPAR-*α* antagonist. Whereas, cotreated cells prevented MCDHFD or PPAR-*α* antagonist-induced morphological changes. This may be the effect of synbiotic on the cell cycle as a stimulator.

#### 3.1.2. Synbiotic Improves the Morphology and Nuclear Condensation of HepG2 and Ameliorates Against PPAR-*α* Antagonist-Induced Damage

**Figure 3:**
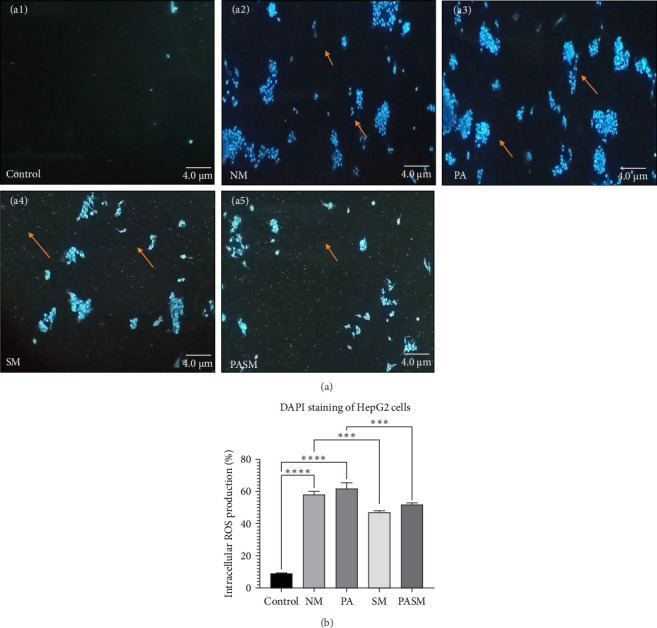
(A) 4-6-Diamidino-2-phenylindole (DAPI) staining of HepG2 cells. (a1) Control cells with normal nuclear characteristics, (a2) nuclear condensation following methionine and choline-deficient high-fat diet (MCDHFD) treatment, (a3) peroxisome proliferators activated receptor (PPAR)-*α* antagonist in HepG2 cells, (a4) SM-treated cells, and (a5) PPAR-*α* antagonist cotreatment with SM. Arrows indicating apoptotic cells, nuclear damage and fragmentation in disease models (NM and PA), and reduced levels in the treated models (SM and PASM). (B) Spectrophotometric analysis were conducted following DAPI incubation at 485 nm. The picture scale bar is 4.0 µm under 40x magnification of a fluorescence microscope. The statistical significance represented as ⁣^*∗∗∗*^*p* < 0.001 and ⁣^*∗∗∗∗*^*p* < 0.0001 of the different groups with respect to disease models as mentioned in Section 2.17 in detail.

The normoxic PA injury was accompanied by a significant inner mitochondrial membrane and nuclear damage, enhanced production of ROS, and apoptosis. The nucleus of cells is fragmented and undergoing chromatin condensation leads to apoptosis. Therefore, observed dense granular spot inside the nuclei of the disease model (Figure 3A, a2), whereas in cells treated with PPAR-*α* antagonist (Figure 3A, a5) and synbiotic combination (Figure 3A, a4) depicts a reduced number of cells, the nuclei fragmentation, reduced chromatin condensation, and less are in early apoptotic phase. These changes in HepG2 cells were detected by fluorescence DAPI stain (Figure 3B). According to these observations as compared to cells of control group (Figure 3A, a1), PPAR-*α* antagonists (Figure 3A, a3) can damage hepatocyte morphology and modulate lipogenesis defects, particularly in the liver.

#### 3.1.3. Synbiotic Alleviate the Formation of Lipid Droplets-Induced ROS in HepG2 Cells

**Figure 4:**
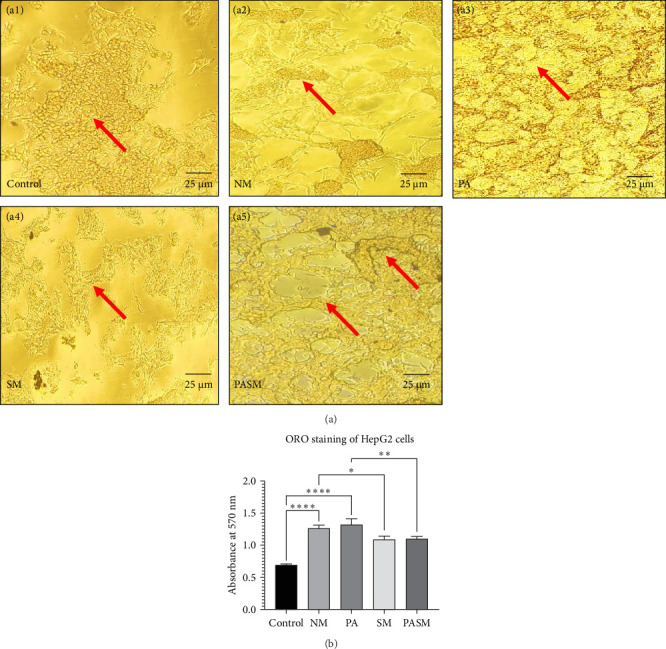
(A) Oil Red O (ORO) stain analysis on HepG2 to check the effect SM on the nonalcoholic steatohepatitis (NASH) model. (a1) Control, (a2) NM, (a3) peroxisome proliferators activated receptor (PPAR)-*α* antagonist-treated cells, (a4) synbiotic, and (a5) PPAR-*α* antagonist cotreatment with SM. Arrows indicating fat depots, apoptotic cells, and lipid accumulation (stained with red). (B) Spectrophotometric analysis ORO stain at 570 nm. The picture scale bar is 25 µm under 40x magnification. The statistical result analysis is demonstrated as ⁣^*∗*^*p* < 0.05, ⁣^*∗∗*^*p* < 0.01, *⁣*^*∗∗∗*^*p* < 0.001, and *⁣*^*∗∗∗∗*^*p* < 0.0001 of the different groups with respect to disease models as mentioned in Section 2.17 in detail.

Liver is the organ responsible for fat depots. As determined by the ORO staining method, treating HepG2 cells exposed to PPAR-*α* antagonist with synbiotic (Figure 4A, a5) or synbiotic (Figure 4A, a4) regulates the lipolysis effectively within the cells as compared to disease model cells (Figure 4A, a2 and a3). HepG2 cells were analyzed microscopically and spectrophotometrically quantified the lipid through the isopropanol extraction method to determine the total lipid accumulation and each group was assayed microscopically at 570 nm. The stained cells of the control group (Figure 4A, a1) generated an absorbance of 0.05 ± 0.002, whereas the absorbance by the PA model was 0.3 ± 0.004, while for cotreatment the value was 0.5 ± 0.010 (Figure 4B). The images obtained signify that the HepG2 cell morphology has been restored when administered with synbiotic.

#### 3.1.4. Synbiotic Reduces PPAR-α Antagonist-Mediated ROS and Inflammation in HepG2 Cells

**Figure 5:**
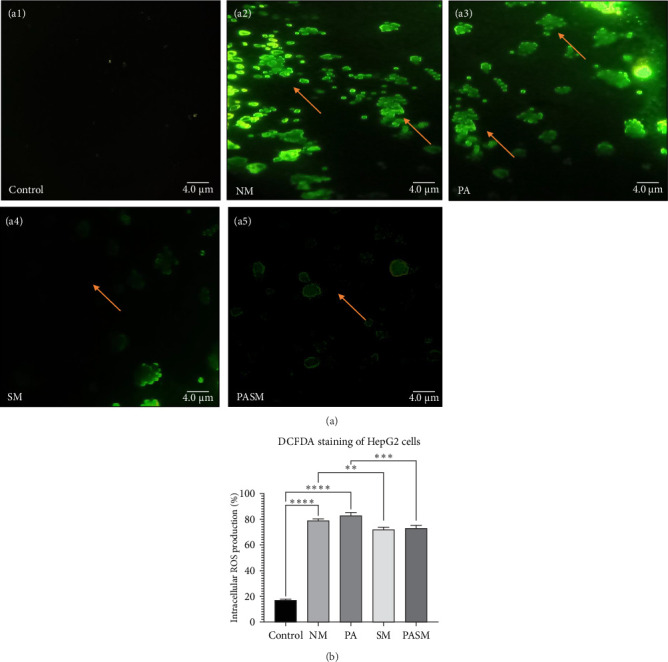
(A) Fluorescent intracellular ROS measurement using 2′-7′-dichlorodihydrofluorescein diacetate (DCFDA) dye in HepG2 cells. (a1) Control; arrow indicating live cells as the nucleus uniform, (a2) nonalcoholic steatohepatitis (NASH) treatment induced stress in HepG2 cells, (a3) peroxisome proliferators activated receptor (PPAR)-*α* antagonist treated cells, (a4) synbiotic combination, and (a5) PPAR-*α* antagonist cotreatment with SM. Arrows indicating ROS formation due to homeostasis break and apoptotic cells. (B) Spectrophotometric analysis of DCFDA stain at 485/525 nm. The data values represent the mean SD of triplicates as *⁣*^*∗∗*^*p* < 0.01, *⁣*^*∗∗∗*^*p* < 0.001, and *⁣*^*∗∗∗∗*^*p* < 0.0001 of the different groups with respect to disease models as mentioned in Section 2.17 in detail. The taken picture scale bar is 4.0 µm under 40x magnification of a fluorescence microscope.

ROS was measured through both fluorescence microscopy using 2′-7′-dichlorodihydrofluorescein diacetate (DCFDA) and the spectrofluorometry method (Figure 5A,B, respectively). The fluorescence microscopic image of cells shows that the green color intensity was seen to be increased 2.5-folds in the disease model (Figure 5A, a2 and a3) as compared to treated (Figure 5A, a4 and a5) and untreated cells (Figure 5A, a1).

#### 3.1.5. Effect of and/or Synbiotic Intervention on the Growth, Morphology, and Barrier Function of Caco2 Cells

**Figure 6:**
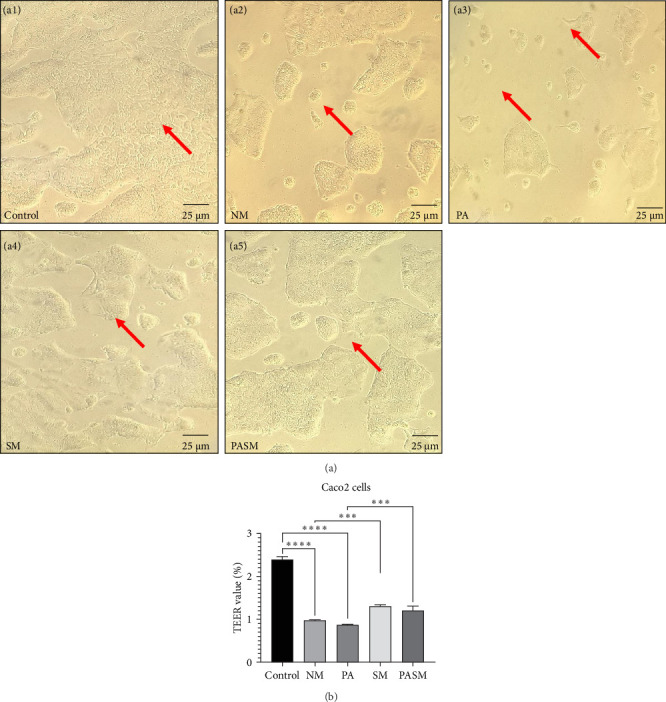
(A) In cell line Caco2 as shown: (a1) Control group (⁣^*∗∗∗∗*^*p* < 0.0001), (a2) NM, (a3) peroxisome proliferators activated receptor (PPAR)-*α* antagonist treated cells, (a4) SM treated cells, and (a5) PA cotreatment with SM (⁣^*∗∗∗*^*p* < 0.001). Arrows indicating loss of membrane structure and deformed intestinal epithelial barrier. (B) Transepithelial electrical resistance (TEER) analysis in %. The picture scale bar is 25 µm under 40x magnification in the microscope. The data presented in mean SD of triplicates as *⁣*^*∗∗∗*^*p* < 0.001 and *⁣*^*∗∗∗∗*^*p* < 0.0001 of the different groups with respect to disease models as mentioned in Section 2.17 in detail.

To observe the morphological changes, Caco2 cells were treated with MCDHFD (Figure 5A, a2). The untreated cells show normal characteristic epithelial morphology (Figure 6A, a1), while the PPAR-*α* antagonist-treated cells (Figure 6A, a2) show constricted cell membranes and distorted cell morphology. Similarly, demorphed and reduced number cells can be observed in PA treated group (Figure 6A, a3). The positive effect on the cell cycle can be seen in cells treated with synbiotic (Figure 6A, a4 and a5). The transition of phases may be induced by the synbiotic. The results obtained suggest that synbiotic combination in Caco2 cells prevents the low level of PPAR-*α* activity (Figure 6A, a5).

#### 3.1.6. Synbiotic Ameliorate the Viability of Caco2 Cells

**Figure 7:**
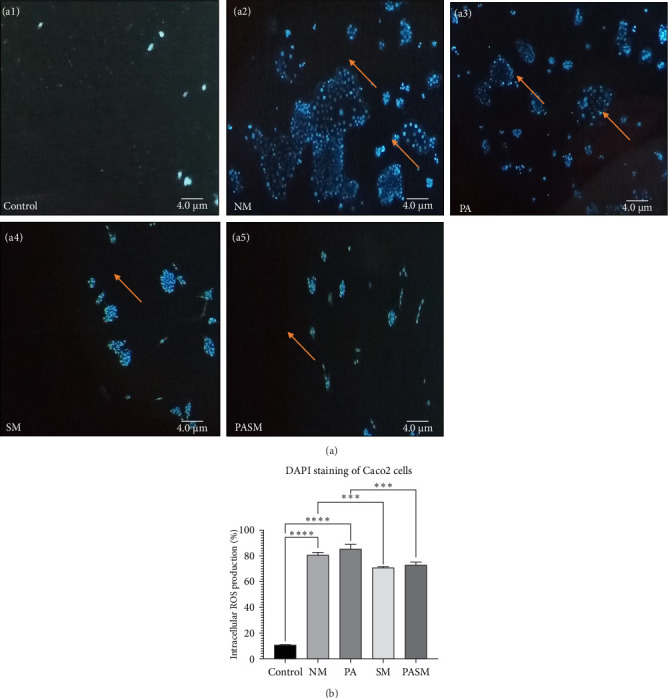
(A) Fluorescent intracellular ROS measurement using 4-6-diamidino-2-phenylindole (DAPI) dye in Caco2 cells. (a1) Control; arrow indicating live cells as the nucleus uniform, (a2) NM, (a3) cells treated with peroxisome proliferators activated receptor (PPAR)-*α* antagonist, (a4) synbiotic combination, and (a5) PPAR-*α* antagonist cotreated with SM. Arrows indicating nuclear condensation in the disease models such NM and PA. Whereas no/reduced levels of apoptotic and damages cells in the groups such as Control, SM, and PASM. (B) Spectrophotometric analyses were conducted following DAPI incubation at 485/525 nm. Data values represent the mean SD of triplicates represented as *⁣*^*∗∗∗*^*p* < 0.001 and *⁣*^*∗∗∗∗*^*p* < 0.0001 of the different groups with respect to disease models as mentioned in Section 2.17 in detail. The picture scale bar is 25 µm under 40x magnification of a fluorescence microscope.

To explore cytotoxic effects of NASH condition on Caco2 cells and on the nucleus specifically, DAPI stain was used. The nucleus of cells is fragmented and undergoing chromatin condensation leading to apoptosis in the disease model group. Therefore, observed dense granular spots inside nuclei. The reduced number of cell nuclei fragmentation, reduced chromatin condensation, and fewer cells were in the early apoptotic phase in the SM group (Figure 7A, a1) as compared to the NASH model (Figure 7A, a2) or PPAR-*α* antagonist group (Figure 7A, a3).

#### 3.1.7. Synbiotic Alleviate the Lipid Accumulation Leading to Barrier Dysfunction in Caco2 Cells

**Figure 8:**
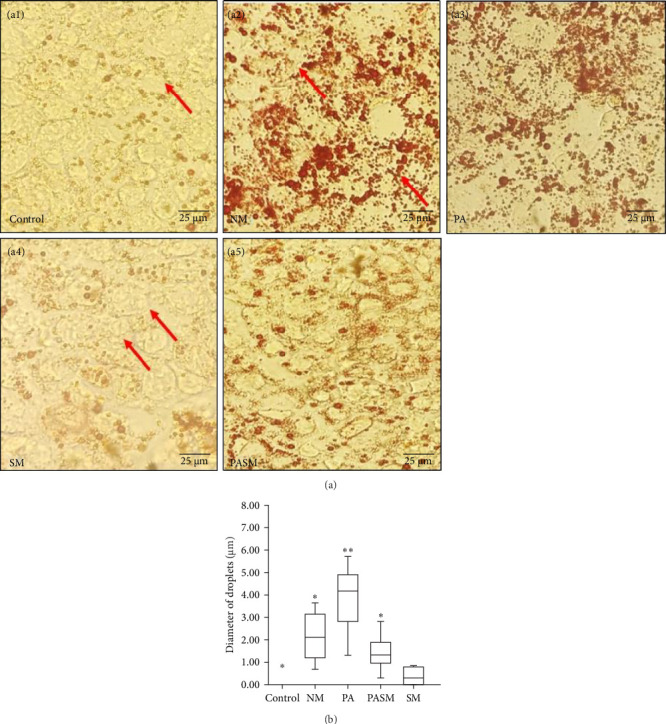
(A) Oil Red O (ORO) stained Caco2 cells. (a1) Control group, (a2) NM, (a3) PA, (a4) SM, and (a5) PASM. Arrows indicating lipid droplets formed in the cells and stained with red. (B) Extraction solvent of ORO absorbance value at 540 nm for lipid droplets of different diameters represented graphically and the picture scale bar is 25 µm under 40x magnification. The statistical result analysis represented as *⁣*^*∗*^*p* < 0.05 and ⁣^*∗∗*^*p* < 0.01 of the different groups with respect to disease models as mentioned in Section 2.17 in detail.

To estimate the PPAR-*α* antagonist impact on Caco2 cells, ORO staining was carried out in the current study. ORO stained Caco2 cells showing fat droplets were formed (Figure 8A, a2 and a3). The fat globules are stained red in the capillary lumen and are indicative of lipid storage dysfunction. Assessment of lipid accumulation is carried out through the number and diameter of the droplets formed of different diameter graphically (Figure 8B). The lipid accumulation was significantly increased in the PA group (Figure 8A, a3) as compared to the control group (Figure 8A, a1). Visible down regulation of deregulated lipogenesis in SM and PASM treated groups (Figure 8A, a4 and a5).

#### 3.1.8. Synbiotic Intervention Reduces MCDHFD and PPAR-*α* Antagonist-Induced ROS Generation and Inflammatory Stress in Caco2 Cells

**Figure 9:**
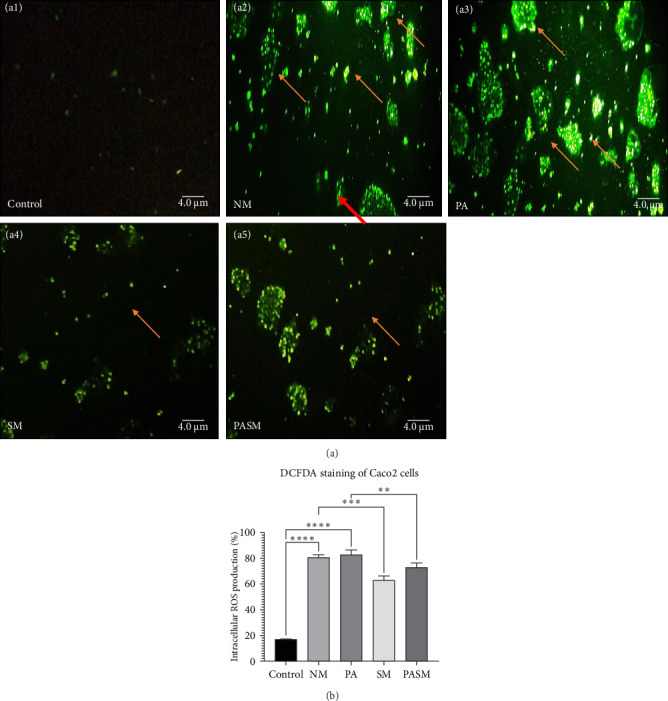
(A) Fluorescent intracellular ROS measurement using 2′-7′-dichlorodihydrofluorescein diacetate (DCFDA) dye in Caco2 cells. (a1) Control, (a2) NM, (a3) SM, (a4) synbiotic combination treated cells, and (a5) PASM. Arrows indicating ROS production in presence or absence of disease and treated models, respectively. (B) Spectrophotometric analysis of Caco2 cells DCFDA stain at 485/525 nm. The data values represent the mean SD of triplicates as *⁣*^*∗∗*^*p* < 0.01, *⁣*^*∗∗∗*^*p* < 0.001, and *⁣*^*∗∗∗∗*^*p* < 0.0001 of the different groups with respect to disease models. The scale bar is 4.0 µm under the fluorescence microscope (40x magnification).

Synbiotic-affected intestinal oxidative stress can be detected by measuring ROS generation in Caco2 cells. A significant increase in ROS generation was observed following PPAR-*α* antagonist stimulation (Figure 9A, a3). However, compared to a PPAR-*α* antagonist treated group, synbiotic combination significantly lowered the ROS accumulation (Figure 9A, a5). This could be happening due to the anti-oxidative properties of AGE on cells, protecting cells from ROS-mediated damages. Similarly, in the disease model cells (NM), there were dense granular stained cells (Figure 9A, a2). Whereas, in cells treated with synbiotic combination (SM), there were fewer fragmented/stained cells, reduced chromatin condensation, and fewer cells in the early apoptotic phase (Figure 9A, a4).

#### 3.1.9. Synbiotic Treatment Reduces the Effects of PPAR-α Antagonist on Lipid Regulator Genes Responsible for ROS Generation and Inflammatory Stress in HepG2 Cells

**Figure 10:**
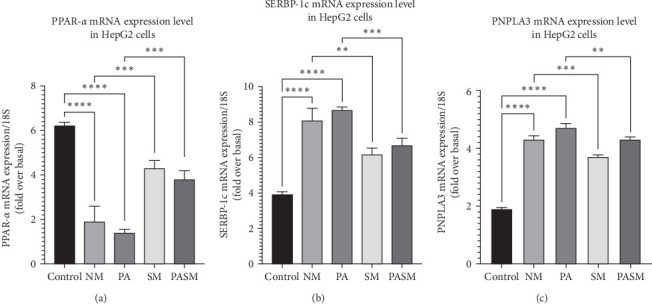
Patatin-like phospholipase domain-containing protein 3/sterol regulatory element binding protein-1c (PNPLA3/ SREBP1-c) pathway qRT-PCR analysis. (A) Peroxisome proliferators activated receptor (PPAR)-*α*, (B) SREBP1-C, and (C) PNPLA3. The data are presented as SD± expressed as ⁣^*∗*^*p* < 0.05, *⁣*^*∗∗*^*p* < 0.01, ⁣^*∗∗∗*^*p* < 0.001, and *⁣*^*∗∗∗∗*^*p* < 0.0001 with respect to disease models as mentioned in Section 2.17 in detail.

PPAR-*α*, PNPLA3, and SREBP-1c levels were assessed to determine the effect of synbiotic on hepatic cells. MCDHFD and PPAR-*α* antagonist promotes aberrant metabolism of lipids in the liver and accrues fatty acid by hepatocellular uptake through de novo biosynthesis and plasma. The mRNA expression (Figure 10) reveals that synbiotic combination effectively and relatively suppresses the expression of causative genes in HepG2 cells.

#### 3.1.10. Synbiotic Improves the PPAR-*α* Antagonist Induced on Dysfunction on Permeability and TJ in Caco2 Cells

**Figure 11:**
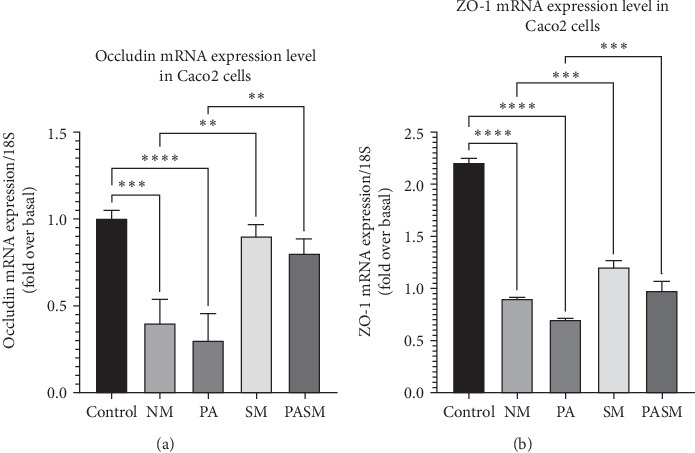
qRT-PCR analysis of barrier function protein: (A) occludin and (B) occluding zone (ZO)-1. All data are presented as SD± expressed as ⁣^*∗*^*p* < 0.05, *⁣*^*∗∗*^*p* < 0.01, ⁣^*∗∗∗*^*p* < 0.001, and *⁣*^*∗∗∗∗*^*p* < 0.0001 with respect to disease models as mentioned in Section 2.17 in detail.

The transcription of occluding and ZO-1 in PPAR-*α* antagonist-treated Caco2 cells was lowered significantly, depicting that the TJs are crucial for gut health. The results of in vitro mRNA expression (Figure 11) indicate that the synbiotic effectively increases the expression of TJs and lowers NASH-mediated inflammation.

#### 3.1.11. Synbiotic Intervention Downregulate the Level of Pro-Inflammatory Cytokines in Hepatic Cells

**Figure 12:**
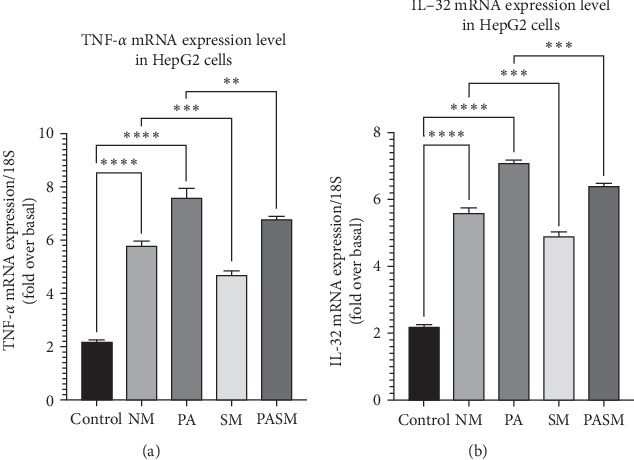
Pro-inflammatory cytokines: (A) TNF-*α* and (B) IL-32. All data are presented as SD± expressed as ⁣^*∗*^*p* < 0.05, *⁣*^*∗∗*^*p* < 0.01, ⁣^*∗∗∗*^*p* < 0.001, and *⁣*^*∗∗∗∗*^*p* < 0.0001 with respect to disease models as mentioned in Section 2.17 in detail.

The MCDHFD in vitro model disrupts the release of inflammatory and pro-inflammatory cytokines in HepG2 cells. This resulted in phenotypic alteration and metabolism in cells. Our results reveal that MCDHFD-fed cells induce high expression of cytokines (Figure 12). Whereas administration with synbiotic lowers the level of pro-inflammatory cytokines such as TNF-*α*.

### 3.2. In Vivo, Synbiotic Interventions Demonstrated a Positive Effect on Liver Tissue

The Table 1 provides data on body weight and liver index for different groups (Control, NM, PA, SM, and PASM). The average body weight for the control group is 415.3 g, while the other groups have slightly lower weights. The liver index, which represents the percentage of liver weight relative to body weight, is also slightly different among the groups, with the control group having the lowest index at 2.4%.

NASH models were determined based on earlier reports [39]. The fibrosis activity with a score ≥5 is diagnosed as NASH and a score ≤3 was diagnosed as no NASH condition. Each rats NASH score was more than 8, as depicted in Table 2.

#### 3.2.1. Synbiotic Reduced Lipogenesis and Accumulation in Rat Hepatic Tissue

**Figure 13:**
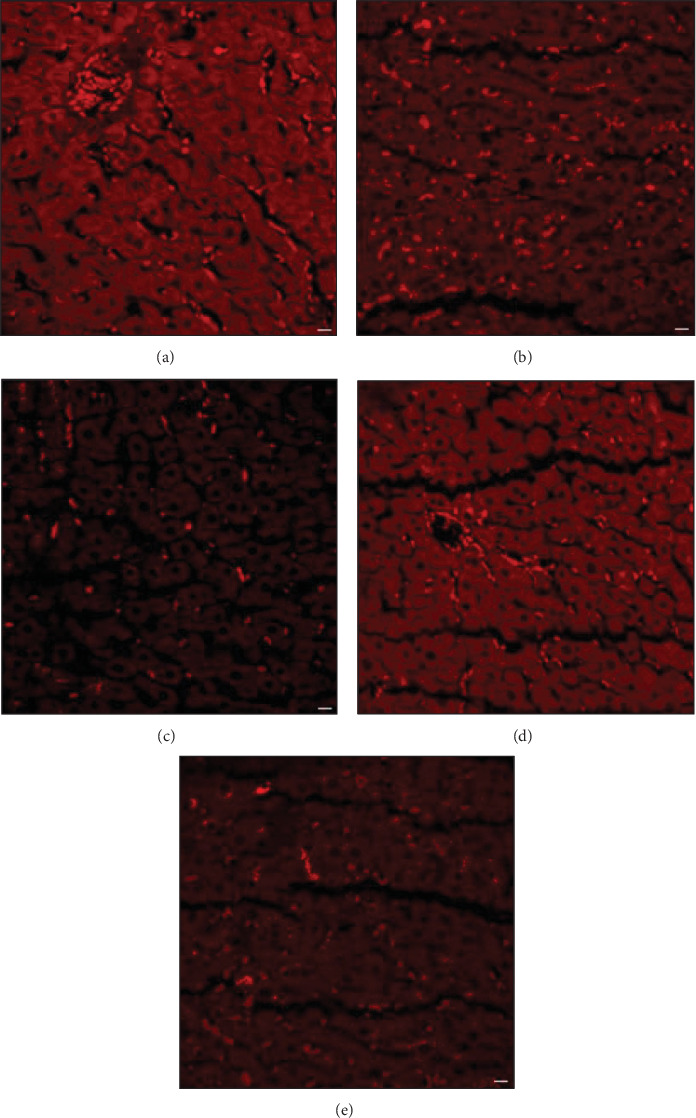
Lipo-genesis stained by Oil Red O (ORO) as standard through a confocal microscope. (A) Control rat liver, (B) NM, (C) SM, (D) PA, and (E) PASM. Phospholipid metabolism deregulation and accumulation is higher in the groups treated with methionine and choline-deficient high-fat diet (MCDHFD; NM) and peroxisome proliferators activated receptor (PPAR)-*α* antagonist (PA) as compared to control and SM treated tissue (described in Section 2.3). Phospholipids stained as fluorescence red droplets in the image can be observed through microscope with scale bar 25 µm under 40x magnification. Representative photographs of livers (fresh) sections, and arrows indicate the hepatic lipid accumulation.

Liver being the crucial organ for fatty acid regulation and metabolism, maintains fatty acid secretion and oxidation within cell and plasma. Therefore, an enhanced level of triglycerides in the system is indicative of fat depots. To assess such findings associated with histological feature changes during NASH condition were observed through confocal imaging and were indicated by arrows. The image shows macrovesicular steatosis, hepatocytes inflammation, and apoptotic bodies' formation.  Figure 13 represents three different groups and the comparative changes in histology (Figure 13A,B). The lipid accumulation within the hepatic cells was reduced in the PASM group (Figure 13E) as compared to the PA (Figure 13C). In the PPAR-*α* antagonist group macrovesicular steatosis can be observed with scattered and distant hepatocytes. The regain of morphology may be due to the efficacy of synbiotic (Figure 13D,E) as a therapeutic agent that can help in lipolysis process through the bioactive compounds present in the AGE.

#### 3.2.2. Rat Colonic Epithelium Lipid Accumulation Reduced Through Synbiotic Intervention

**Figure 14:**
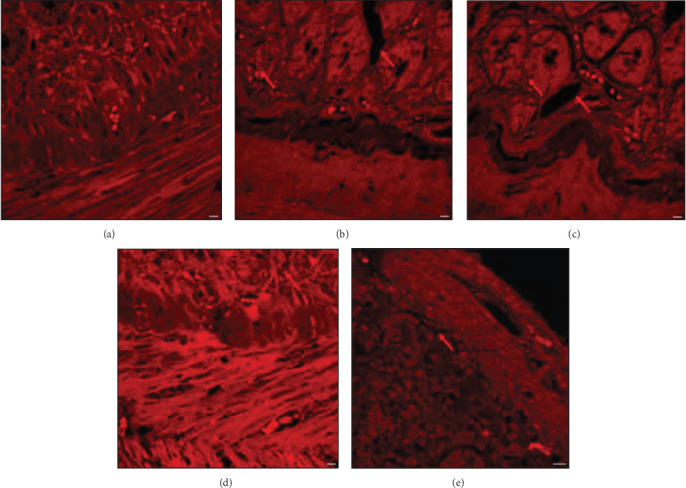
Confocal Oil Red O (ORO) staining of fresh gut samples of rats nonalcoholic steatohepatitis (NASH) model. (A) Control model, (B) NM, (C) PA model, (D) SM, and (E) PASM. Stained are the lipid droplets or phospholipids. Peroxisome proliferators activated receptor (PPAR)-*α* antagonist cotreatment with SM administration lead to colon epithelial layer disruption, while regained and improved barrier function and morphology after treatment with synbiotic intervention (represented by arrows). The image can be observed through microscope with scale bar 25 µm under 40x magnification.

In the PPAR-*α* antagonist group (Figure 14C), the ORO staining revealed the development of lipid droplets in the colon bilayer along with crypts of Lieberkuhn. In an anomalous shape, TJs are disrupted. Comparing the control group (Figure 14A) with the NM group (Figure 14B), synbiotic treatment (Figure 14D) shows improvements in epithelial barrier function and reduced lipogenesis. The antioxidative and anti-inflammatory properties of the prebiotic and synergistic effect of probiotic may be responsible for the reduced effect of PPAR-*α* antagonist in the PASM group (Figure 14E).

#### 3.2.3. Synbiotic Effect on MCDHFD and PPAR-*α* Antagonist-Induced Decrease in Hepatic Cell Dimensions, Hepatocytes Mass, and Lipid Aggregation in Wistar Rat Model

**Figure 15:**
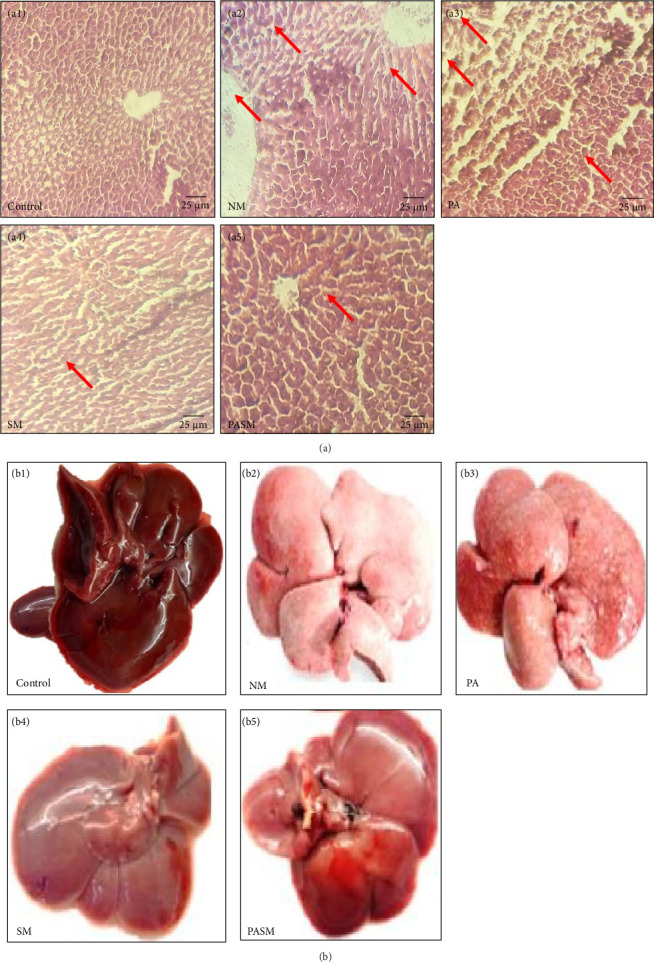
(A) Hematoxylin and eosin (H/E) staining of liver tissue. (a1) Control group, (a2) NM, (a3) PA, (a4) SM, and (a5) PASM. Arrows indicating presence or absence of hepatocytes degeneration, scattered lobular inflammation in disease model, or treated model, respectively. (B) (b1–b5) Lipid accumulation in male Wistar rat liver model depicted by red arrows. Six rat liver samples were analyzed for the experiment.

The histologic characteristics of NASH model (Figure 15A, a2) and PPAR-*α* antagonist (Figure 15A, a3) shown here are indicated by the arrow. The changes reported as compared to the control group (Figure 15A, a1) are steatosis of macrovesicles hepatocyte ballooning, inflammation, and clear fibrosis. Furthermore, Mallory bodies and apoptotic bodies formation were also reported (Figure 15A, a1 and a2). H/E staining of liver tissue of PA rats (Figure 15A, a3) shows Mallory body formation due to hepatocyte degeneration and intralobular inflammatory infiltrations of mononuclear cells, whereas in the PASM (Figure 15A, a5) and SM group (Figure 15A, a4), treated hepatocytes microvesicular degeneration and diffusion was reduced.

#### 3.2.4. Synbiotic Ingestion in Wistar Rats Regulates MCDHFD Induces Modifications in Colon Tissue and Integrity

**Figure 16:**
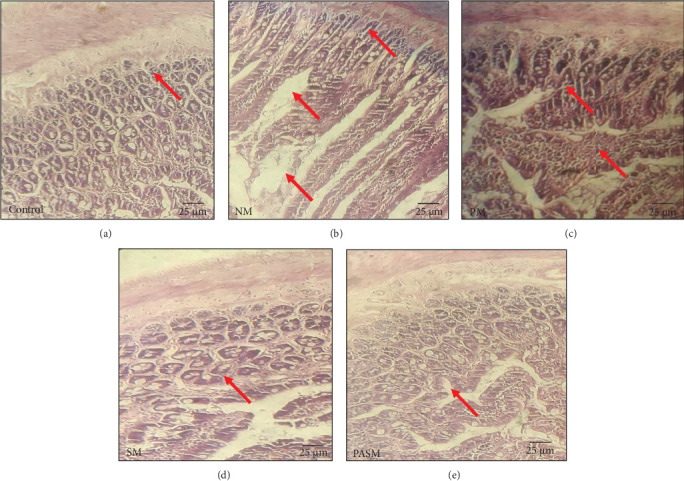
Histopathological changes detected by hematoxylin and eosin (H/E) staining of rat's colon. (A) Control, (B) NM, (C) PA, (D) SM, and (E) PASM. The disease development and morphological changes are marked by arrows.

The lipid droplets and colonic epithelium assayed through H/E staining in the NM and PA (Figure 16B,C) show colon epithelial layer disruption and regained morphology of epithelial barrier in the SM treatment group (Figure 16D,E). The anomaly in epithelium, crypts distortion reduction in the crypts of Lieberkuhn, and alteration in colon mucosa are indicatives of PPAR-*α* antagonist-affected gut barrier function with respect to the histology of normal group (Figure 16A). This suggests that the combination rectifies the defects caused by antagonist.

#### 3.2.5. The Effects of Synbiotic on Lipid Profiles in Rodents With NASH

**Figure 17:**
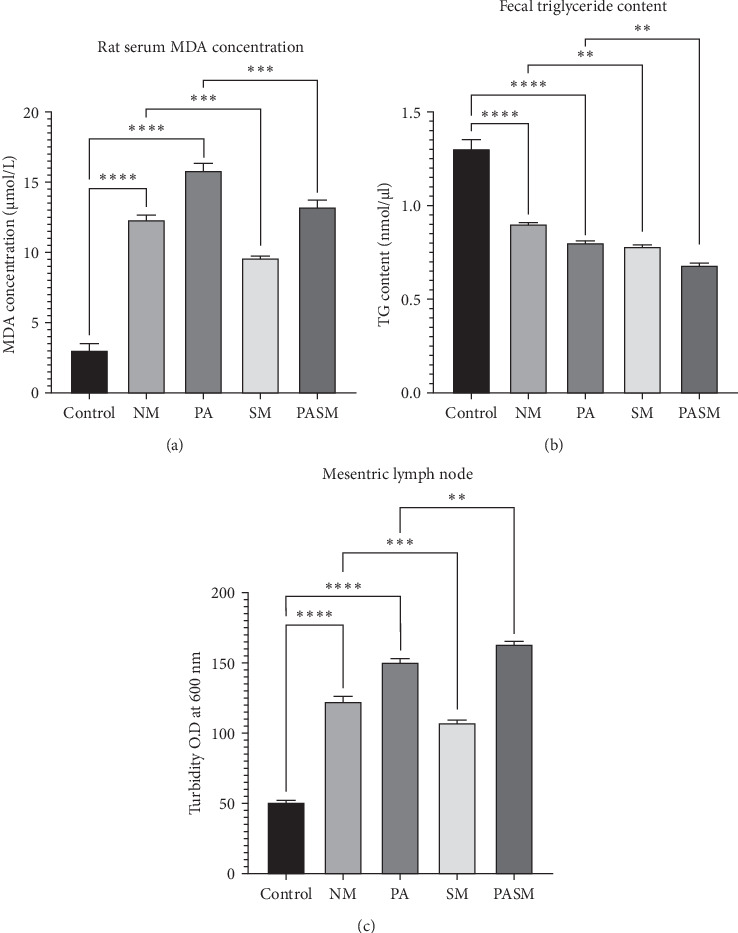
Effect on gut (A) rat colon malondialdehyde (MDA) concentration: (a1) control, (a2) disease control, (a3) PA, (a4) synbiotic model, and (a5) cotreated model. (B) Fecal triglyceride content and (C) mesenteric lymph node (MLN) colonization detected was reduced in the synbiotic treated rats' model as compared to disease model. Where mean SD± represented as ⁣^*∗*^*p* < 0.05, *⁣*^*∗∗*^*p* < 0.01, *⁣*^*∗∗∗*^*p* < 0.001, and *⁣*^*∗∗∗∗*^*p* < 0.0001 with respect to disease models as mentioned in Section 2.17 in detail.

**Figure 18:**
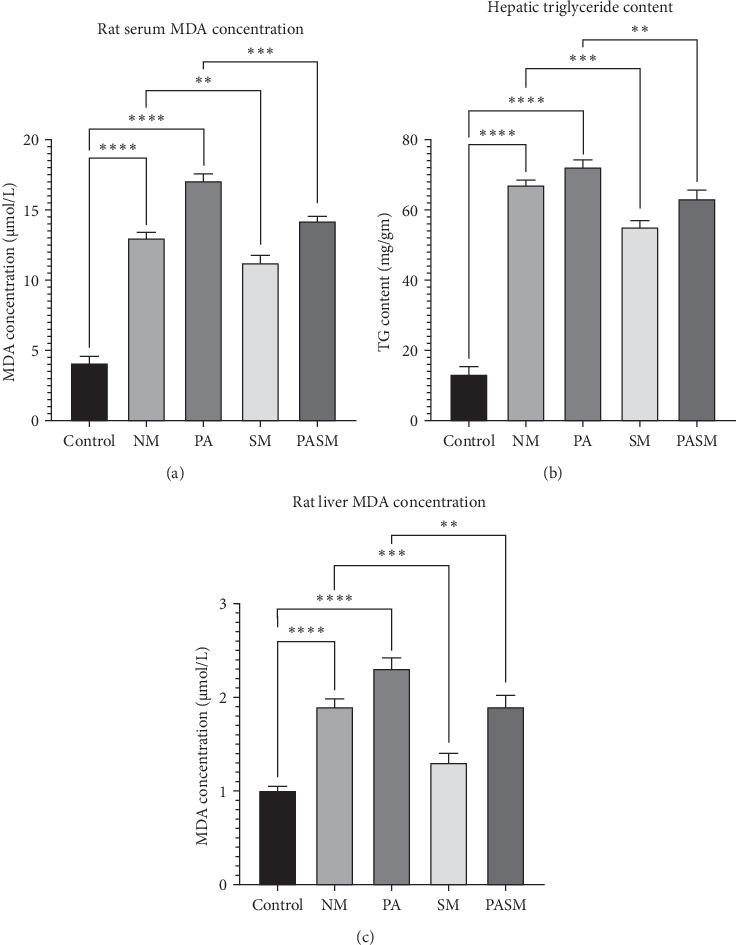
(A) Rats' serum malondialdehyde (MDA) levels, (B) liver triglyceride content, and (C) liver MDA levels. The mean SD± demonstrated as *⁣*^*∗*^*p* < 0.05, *⁣*^*∗∗*^*p* < 0.01, *⁣*^*∗∗∗*^*p* < 0.001, and *⁣*^*∗∗∗∗*^*p* < 0.0001 with respect to disease models as mentioned in Section 2.17 in detail.

The lipid profile analysis showed that PA rat showed elevated levels of colon malondialdehyde (MDA), fecal TG, and mesenteric translocation (Figure 17A, B and –C) as compared to controls. Conversely, HDL-C levels in the serum were low. The treated groups with synbiotic combination comparatively showing reduced levels of lipid profiles. The lipid profile research also reveals a statistically discernible difference in the levels of serum, hepatic triglycerides, and rat liver MDA, sample between the groups (Figure 18A, B and –C, respectively).

#### 3.2.6. Synbiotic Regulate the PPAR-*α* Antagonist-Induced Lipid Regulatory Genes, Pro-Inflammatory, and Other Related Cytokines Responses of Hepatic Tissue and Improves Anti-Inflammatory Activities

**Figure 19:**
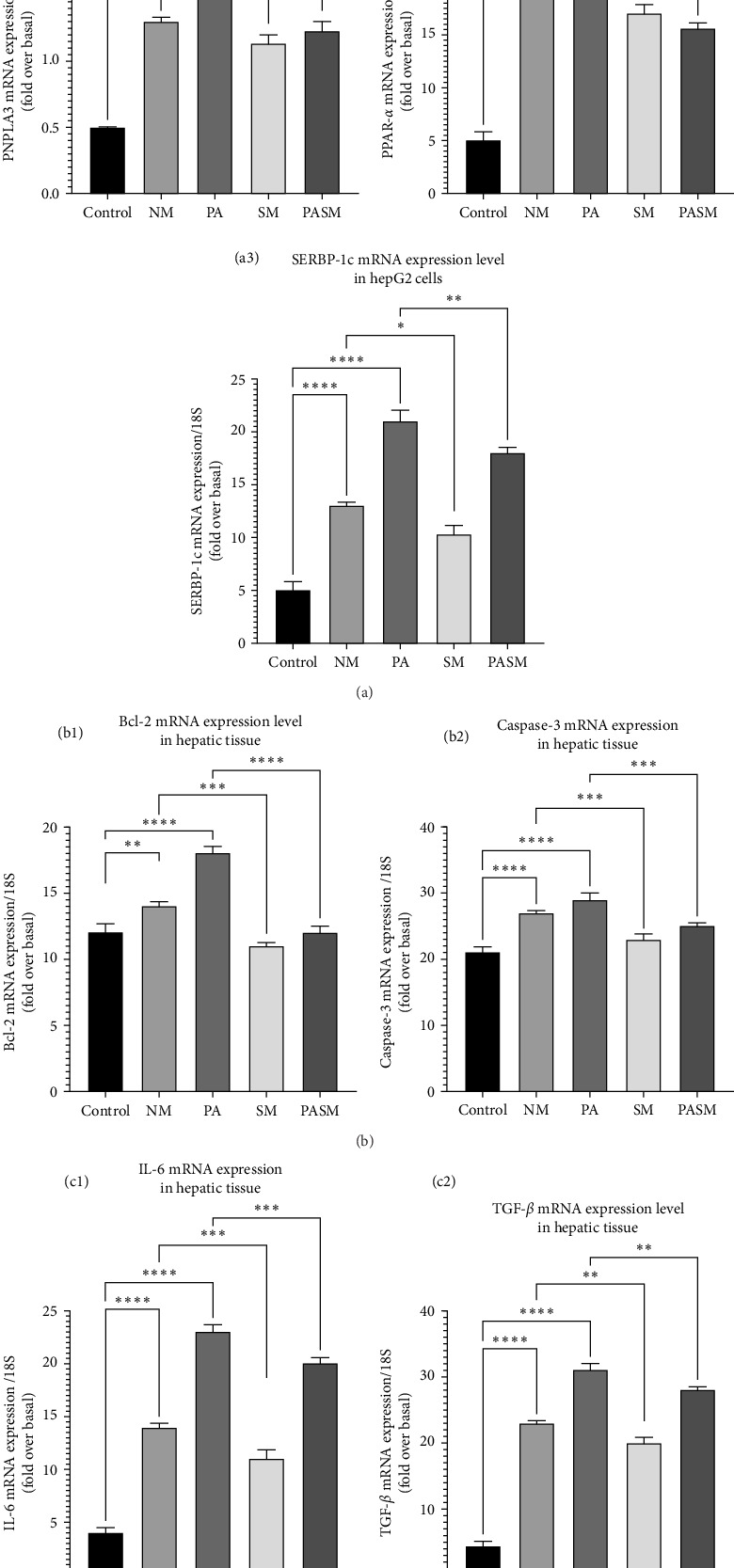
The qRT-PCR analysis of (A) pathway genes (a1) patatin-like phospholipase domain-containing protein 3 (PNPLA3), (a2) peroxisome proliferators activated receptor (PPAR)-*α*, and (a3) sterol regulatory element binding protein-1c (SREBP-1c); (B) apoptotic factors (b1) Bcl-2 and (b2) caspase-3; and (C) cytokines (c1) IL-6 and (c2) TGF-*β*. The data presented here as mean SD± expressed as *⁣*^*∗*^*p* < 0.05, *⁣*^*∗∗*^*p* < 0.01, *⁣*^*∗∗∗*^*p* < 0.001, and *⁣*^*∗∗∗∗*^*p* < 0.0001 with respect to disease models as mentioned in Section 2.17 in detail.

In NASH condition led by PPAR-*α* antagonist disrupts the release of lipid regulatory genes, and pro-inflammatory and inflammatory cytokines from liver and gut tissue resulting in the deregulation of hepatic metabolism and phenotypic changes [6, 11]. The rat NM and PA models revealed an increase of PNPLA3, SREBP-1c, and decrease of PPAR-*α* expression (Figure 19A, a1–a3). Similarly, the apoptotic gene regulators expression also improves in the presence of SM (Figure 19B, b1 and b2). Moreover, after synbiotic intervention, there was a simultaneous downregulation registered of cytokines such as IL-6 and TGF-*β* (Figure 19C, c1 and c2).

#### 3.2.7. Synbiotic Alleviate the Protein Levels of PNPLA3 and TGF-*β* Suggesting Improved Lipid Regulation and Epithelial–Mesenchymal Transition/Changes (EMT) in Hepatic Cells

**Figure 20:**
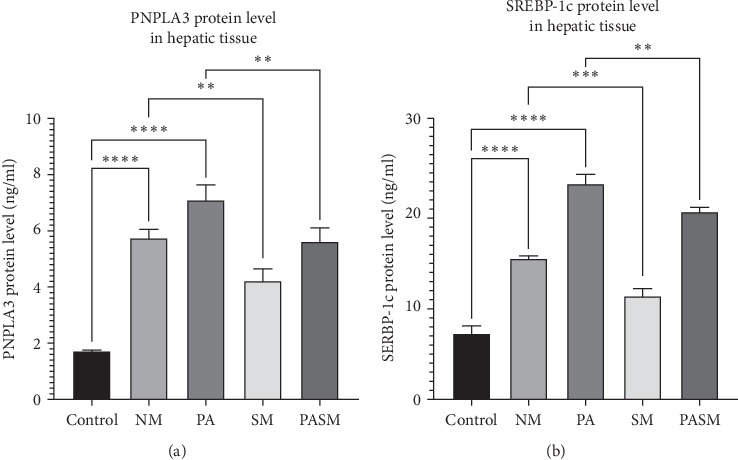
Protein levels of (A) patatin-like phospholipase domain-containing protein 3 (PNPLA3) and (B) sterol regulatory element binding protein-1c (SREBP1-c) in hepatic tissue were measured using enzyme-linked immunosorbent assay (ELISA). The livers of six rats from each group were analyzed. Values are expressed as ±mean SD± represented as *⁣*^*∗*^*p* < 0.05, *⁣*^*∗∗*^*p* < 0.01, *⁣*^*∗∗∗*^*p* < 0.001, and *⁣*^*∗∗∗∗*^*p* < 0.0001 with respect to disease models as mentioned in Section 2.17 in detail.

Rodents with NASH condition and treated with PPAR-*α* antagonist showed histopathological changes and the epithelial nature of hepatic tissue changes to fibrotic and elongated in phenotype (Figure 15B). Possibly due to overexpression of PNPLA3 parenchymal and nonparenchymal cell interactions starts and may affect features of metabolic disorder through lipid accumulation and modulating fat content in NASH condition. This causes a cascade of signaling that is initiated by high amounts of TGF-*β* and low levels of PPAR-*α*. These EMT affect the morphology of liver cells. This upregulation of TGF-*β* induced by PPAR-*α* antagonist activates the downstream transcription factors signaling causing various modifications in hepatic tissue. It is reported previously that TGF-*β* plays an inevitable role in NASH pathogenesis. TGF-*β* induces fibroblast marker up regulation such as fibronectin-1, alpha-smooth muscle actin (*α*-SMA), and epithelial E cadherin downregulation. Synbiotic treatment shows downregulation of PNPLA3 and TGF-*β* through the PPAR-*α* pathway demonstrated in protein profile analysis (Figure 20A,B).

#### 3.2.8. Functional Pathway Enrichment Analysis of AGE

**Figure 21:**
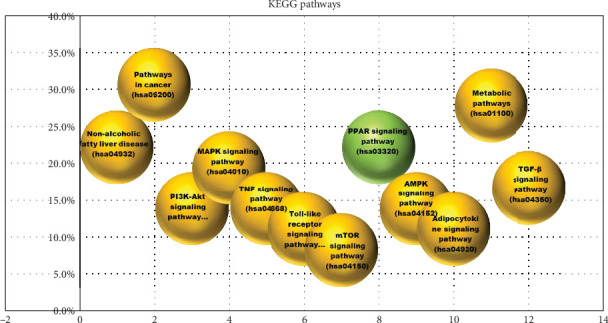
Kyoto Encyclopedia of Genes and Genomes (KEGG) pathway enrichment analysis. The *X*-axis shows the pathways, whereas *Y*-axis indicates the % of pathways hit by aged garlic extract (AGE) and high expression protein genes were significantly enriched in pathways. It is found that 12 common adjustment pathways are present through KEGG analysis with *p* < 0.05 significance. The green color indicates the target pathway, while other affected pathways are represented with yellow color.

KEGG has been used for performing analysis of bioactive agents associated with NASH condition targets to explore their functions and efficacy inside the human body (Figure 21).

#### 3.2.9. AGE Possess Inhibition Affinity for PNPLA3 as a Target Protein for NASH Prevention

**Figure 22:**
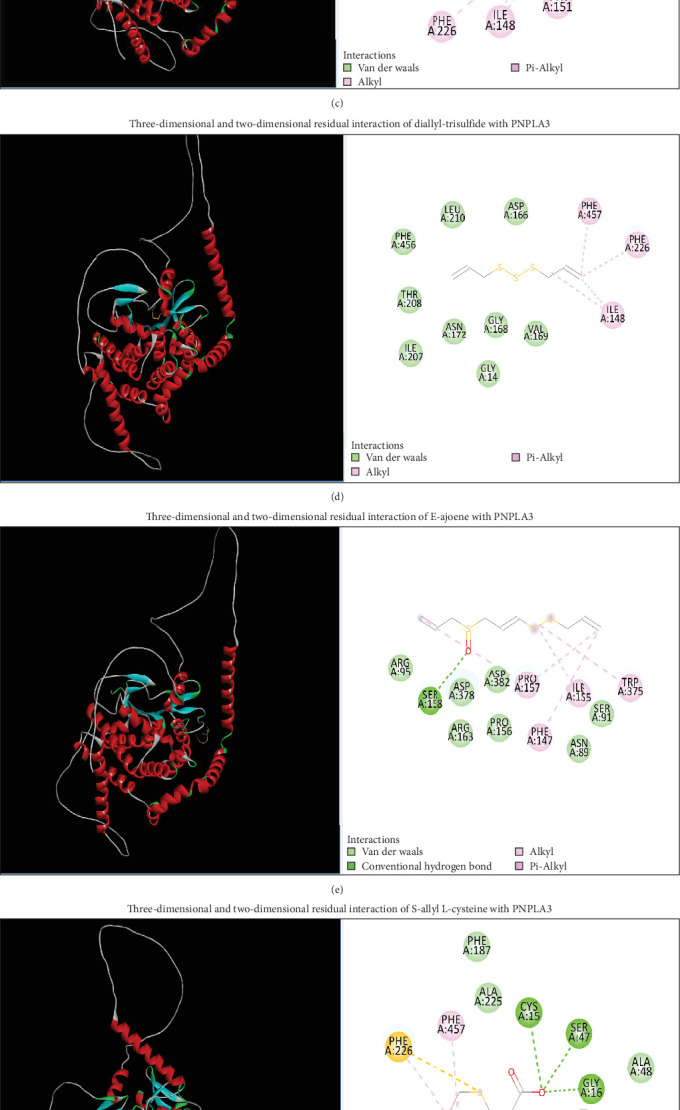
MD of eight bioactive components of aged garlic extract (AGE) with patatin-like phospholipase domain-containing protein 3 (PNPLA3). 3D and 2D interactions of (A) allicin (B) diallyl sulfide, (C) di-allyl disulfide, (D) diallyl tri-sulfide, (E) E-ajoene, (F) S-allyl-L-cysteine, (G) S-allyl cysteine sulfoxide, and (H) Z-ajoene.

Several pro-inflammatory cytokines are produced by irregular diet, leading to oxidative stress in the body. PNPLA3 inhibition prevents different cytokines and inflammatory reactions, which may play a role as a protective compound that attenuate the NASH progression. In the field of computational biology, docking is a method which may be used to predict ligands' experimental binding modes and their relative affinity when bound to target receptors through their binding pockets. C-D-T network interactions and binding energy were analyzed using molecular docking for bioactive components from AGEs. The bond affinities of S-allyl cysteine sulfoxide, S-allyl-L-cysteine diallyl-disulfide, allicin, Z-ajoene, diallyl sulfide, diallyl tri-sulfide, and E-ajoene with PNPLA3 were −5.4, −5.2, −4.0, −4.3, −4.6, −3.9, −3.7, and −4.8 kcal/mole, respectively. These eight bioactive components of AGE expressed binding affinity for PNPLA3, suggesting that AGE as a compound may target PNPLA3 as a core protein to reduce the NASH progression as shown (Figure 22).

## 4. Discussion

Pathogenesis of progressive NASH is primarily driven by oxidative stress and inflammation [35]. A high fat diet, as well as abnormal metabolism and hereditary variables such as polymorphism genes, may all contribute to the advancement of NASH, gut dysbiosis, lining eruption, and microbial translocation [39]. Persistent excessive lipid metabolism deregulation has an adverse effect on liver and colon tissue and affects host health negatively [40]. There is an absence of FDA-approved pharmacological or dietary treatment available to prevent or cure NASH. It may be prevented by liver transplant. There are few treatments available till the fibrosis stage. One tailored therapy on *L. rhamnosus*, as a probiotic improves intestinal integrity [41]. This investigation on the prevention of NASH progression by AGE and *L. plantarum* MTCC 2621 is the first to demonstrate the preventive efficacy of this combination. SREBP-1c is a crucial gene implicated in NASH development and progression, cell cycle control, apoptosis, lipid metabolism, and transcription regulation, among other functions and has widely been associated with steatosis in many studies [41, 42]. PPAR-*α* has an important role in various activities of physiology such as glucose homeostasis, lipid metabolism, and anti-inflammatory action [43]. Similarly, PNPLA3 is associated with hepatic lipid accumulation and eventually contributes to the NASH progression. Meanwhile, there is no direct link between SREBP-1c association with cholesterol homeostasis still the levels of cholesterol get modulated in the NASH condition and PPAR-*α* activation/deactivation [44, 45].

This article describes a series of experiments to elucidate the function of PPAR-*α* and PNPLA3 in the pathogenesis of NASH, which results in hepatic tissue damage. In vitro studies were conducted on synbiotic combinations that showed no cytotoxicity on HepG2 cells with 180 mg/kg concentration of AGE and 10^9^ CFU/mL concentration of probiotic till 48–72 h. At the cellular level, synbiotic combination enhanced the growth rate of HepG2 cells treated with PPAR-*α* antagonist concerning other groups with no AGE or synbiotic combination as demonstrated (Figure 2; *p* < 0.001). To study such intracellular oxidative stress, a lipophilic and nonfluorescent compound DCFDA has been used where PPAR-*α* antagonist model cells showed more prominent green fluorescent as compared to control and cosupplementation of synbiotic resulted in a significant decrease in oxidative stress as apparent by weak fluorescence with respect to NM group. The apoptosis term refers to peculiar cell death (Figures 5 and 9). In this study, nuclear morphology was examined using DAPI dye to assess apoptotic changes. The cells treated with PPAR-*α* antagonist show visual evidence of nuclear condensation with respect to cells of the normal group. A significant inhibitory effect of synbiotic treatment was observed on hepatic and colon cell apoptosis. The rate of apoptosis in the PASM group was reduced than in the PA (*p* < 0.001) for NASH treatment (Figures 3 and 7).

To investigate whether the synbiotic combination protects against NASH mediated by PPAR-*α* antagonist condition by mediating intestinal barrier disruption of the mucosal membrane the permeability of Caco-2 cells and rat colons was examined. Regained percentage of TEER (%) and intestinal barrier function indicates the positive effect of the synbiotic combination (Figure 6B). ZO-1 is required for the integrity of transmembrane proteins such as occluding, where occluding supports the structural integrity and membrane barrier function [45]. In support of previous research, Occludin and ZO-1, components of TJ assembly, were reduced in the disease model. However, in the treated gut tissue, this function was regained (Figure 10A).

ROS produced during lipid metabolism dysfunction leads to hepatic apoptosis. To verify the same, Bcl-2 and caspase3 could be good apoptotic biomarkers. The reduction in the ROS formation in synbiotic-treated cells unravels the preventive effect of the combination on cells (Figures 17 and 18). Studies in previous reports stated that *n* = 3 chain polyunsaturated fatty acid consumption lowered the ratio of SREBP1-c to PPAR-*α* [46]. This ratio change contributes to fatty acid oxidation and steatosis [47]. In this study, PA rats resulted in increased fat mass, leaky gut, and liver inflammation, as well as insulin resistance, which is linked to fatty acid overflow and the buildup of hepatic fat. In addition to gut barrier dysfunction, AMPs production, immune function, and TJ accessibility inhibit bacterial translocation simultaneously [48, 49]. The increased levels TGF-*β* modulates intracellular oxidative pathways in vascular tissues such as the liver to affect cell adhesion of cells, proliferation, and differentiation leading to apoptosis [50, 51].

Moreover, the treatment with PPAR-*α* antagonist on rats' liver resulted in oxidative stress elevation, which leads to lipid peroxidation. Lipid peroxidation results in the MDA formation and oxidative stress marker in the hepatic cells. The exacerbated MDA levels were measured. Clear amelioration is visible after synbiotic treatment. Further, these reduced levels of MDA in the tissue were measured through fluorescence spectroscopy. The confocal images of the liver and gut stained with ORO correlated with restored membrane and barrier function along with mRNA expressions, which is crucial for the prevention of NASH and translocation of bacteria (Figures 12 and 13).

PASM was found to express lower levels of inflammatory cytokine mRNA compared to the PA group (*p* < 0.01; Figure 19A, a2), suggesting the cumulative effect of the symbiotic combination also improved the PPAR-*α* levels. *Lactobacillus plantarum* administration may contribute to the modulations associated with the human gastrointestinal tract. Moreover, to support the theory results of protein levels of PNPLA3 and SREBP1-c also have been checked. The clear drop in the protein levels of PNPLA3 and SREBP1-c suggests the role of AGE in targeting the said pathway (Figure 20).

The KEGG enrichment analysis resulted in the metabolic pathways (hsa01100) being the most affected in the presence of AGE. Other signaling pathway may be affected by the presence of AGE. SM reduces the inhibition of PPAR-*α* and delays the progression of NASH by reducing inflammatory responses. In molecular docking, ligands binding affinities obtained were −5.4, −3.7, −4.0, −3.7, −4.2, −4.8, −5.2, and −4.8 kcal/mole, of S-allyl cysteine sulfoxide, diallyl sulfide, diallyl-disulfide, diallyl trisulfide, allicin, E-ajoene, Z-ajoene, and S-ally, respectively. S-allyl cysteine sulfoxide binds and downregulates PNPLA3 proteins more efficiently. All eight bioactive components are depicted (Figure 22) with their hydrogen and hydrophobic interactions and distances from the PNPLA3 receptor. The negative binding affinity of these components indicates that they can bind to the PNPLA3 core protein.

## 5. Conclusion

**Figure 23:**
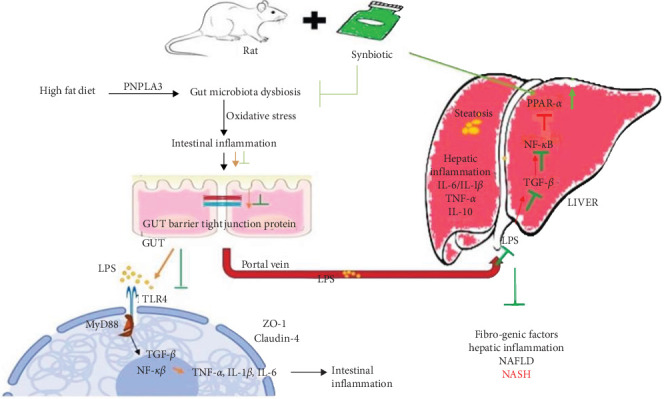
Demonstration of synbiotic-mediated activation of peroxisome proliferators activated receptor (PPAR)-*α* leading to the downregulation of patatin-like phospholipase domain-containing protein 3/sterol regulatory element binding protein-1c (PNPLA3/SREBP1-c)-induced cytokines contributing to liver cell inflammation.

In conclusion, the current study suggests that the synbiotic has synergistic preventive effects on the development and progression of NASH. The synbiotic combination used can reduce liver tissue injuries and gut dysbiosis mediated by the high expression of PNPLA3 and SREBP-1c and improves the activity of PPAR-*α* (Figure 23). The results enhanced the antioxidant enzyme levels to eradicate the free radicals, protected hepatocytes, and lowered lipid peroxidation. In the form of food additives, clinical translation of this study would be beneficial to NASH human subjects.

## Figures and Tables

**Table 1:** Rat body weight and rat liver tissue weight.

Parameters	Control	NM	PA	SM	PASM
Body weight (g)	415.3 ± 43.5	250 ± 20	236 ± 0.7	361 ± 9	358 ± 30.7
Liver index (%)	2.4 ± 0.5	2.51 ± 0.5	2.7 ± 0.5	3.5 ± 0.4	3.1 ± 0.5

**Table 2:** The score of the study set.

Events	Gradation	Percentage of occurrence (%; *n* = 30)	Score
Hepatic steatosis gradation	Liver parenchymal steatosis	—	—
	<5%	0	0
	5%–35%	2	1
	>36%–62%	29	2
	>63%	69	3

Fibrosis stage	None	43	0
	Phase 1, pericellular/sinusoidal; zone 3	7	1
	Phase 2, perisinusoidal and portal; zone 3	23	2
	Porto-central (panacinar) fibrosis	4	3
	Cirrhosis	5	4

Nonzonal patches (contiguous patches)	Microvesicles	81	0
	Not present	19	1
	Present	—	—

Nodular inflammatory foci	Foci absent	0	0
	<2/200 × field	1	1
	2−4/200 × field	57	2
	>4/200 × field	42	3

Large lipogranulomas	Absent	71	0
	Present	29	1

Micrograulomas	Absent	61	0
	Present	39	1

Liver injury and ballooning	No liver injury	23	0
	No ballooning	58	1
	Few prominent ballooning of cells present	19	2

Portal inflammation	Absent	51	0
	Greater than minimal	49	1

Macrophages activation	None/few	91	0
	Many	15	1

Hyaline of Mallory bodies	None/few	65	0
	Many	35	1

Glycogenated cell's nuclie	Absent	51	0
	Present	49	1

Acidophil cells	Absent	89	0
	Many	11	1

Giant mitochondria	None/few	73	0
	Many	26	1

Diagnostic classification	Not staetohepetitis	0	0
	Boaderline	1	1
	Prominent steatohepetistis	99	2

## Data Availability

The data that supports the findings of this study are available in the supporting information of this article.

## Supporting Information

Supporting Information Figure S1. Bacterial growth curve: *Lactobacillus plantarum* MTCC 2621 proliferation in MRS broth with and without AGE. Tables S1 and S2. List of primers (*Mus musculus* and human, respectively) and Tables S3 and S4 listing ingredient/nutrient and vitamins composition of the control group Wistar rat diet (g/kg) are shared as Supporting data and are available online.
