# Supplementary material for: In Vitro, In Vivo, and In Silico Investigation of Synbiotic-Mediated Activation of PPAR-α Curtails Nonalcoholic Steatohepatitis (NASH) in Wistar Rats by Inhibiting PNPLA3/SREBP1-c Lead Inflammatory Injury of Hepatic Cells
Source: Mediators Inflamm. 2025 Feb 19;2025:9948679. doi: 10.1155/mi/9948679 (PMC11865469; doi:10.1155/mi/9948679)
Supplement: Supporting Information — Figure S1. Bacterial growth curve: Lactobacillus plantarum MTCC 2621 proliferation in MRS broth with and without AGE. Tables S1 and S2. List of primers (Mus musculus and human, respectively) and Tables S3 and S4 listing ingredient/nutrient and vitamins composition of the control group Wistar rat diet (g/kg) are shared as Supporting data and are available online. [file 9948679.f1.docx]

**Supplementary Material 1**

**Bacterial growth on De Man, Rogosa and Sharpe medium (MRS) in presence of aged garlic extract**

**Supplementary Figure 1**: Bacterial Growth Curve: *Lactobacillus plantarum* MTCC 2621 in MRS broth. The presence of AGE resulted in an approx. 6 hour early log phase when compared to the absence of AGE. Garlic extract added to artificial growth feed can significantly increase (*P*<0.01) the growth.

**Supplementary Table 1**: List of *Mus musculus* primers utilized during quantitative real-time polymerase chain reaction (PCR) process

| **Name** | **Primer** | **Type** |
| --- | --- | --- |
| **PNPLA3** | TCTCCCTCTCGATCACATCAT  CCATCGGACACTCTGGTGAG | Forward  Reverse |
| **SREBP-1c** | CGCTTCTTACAGCACAGCAAT TGCCCAAGGACAAGGGGCTA | Forward  Reverse |
| **PPAR-** α | GTCAGCTGCCCTGCTGTCCCA CGAAAGAAGCCCTTGCAGCC | Forward  Reverse |
| **IL-6** | GCCCTTCAGGAACAGCTATGA  TGTCAACAAATCAGTCCCAAG | Forward  Reverse |
| **Caspase-3** | GTGGAACTGACGATGATATGGC  CGCAAAGTGACTGGATGAACCG | Forward  Reverse |
| **TNF- α** | GTGGCGGGGGCCACCACGCTC CGAGTTTTGAGAAGATGATCG | Forward  Reverse |
| **TGF-β** | TGCTTCAGCTCCACAGAGAA  TGTGTTGGTTGTAGAGGGCAT | Forward  Reverse |
| **Bcl-2** | GTGCACCGAGACACGGCTGC CGACGGTAGCGACGAGAGAA | Forward  Reverse |
| **IL-32** | TCAAAGAGGGCTACCTGGAGAC  TCTGTTGCCTCGGCACCGTAAT | Forward  Reverse |
| **Occluidn** | CTCGAGAAACTGAGTGCCTGGAC  AAGCTTTCGACCAATTCACCTGA | Forward  Reverse |
| **ZO-1** | ACTATGGCACATCAGCACG  TGGGCAAACAGACCAAGC | Forward  Reverse |
| **18S** | ACGGAAGGGCACCACCAGGA  CACCATCACCTACGGAATCG | Forward  Reverse |

**Supplementary Table 2**: List of Human primers utilized during quantitative real-time PCR process.

| **Name** | **Primer** | **Type** |
| --- | --- | --- |
| **PNPLA3** | CGAGGCGAGCGGTACGT  TGACACCGTGATGGTGGTTT | Forward  Reverse |
| **SREBP-1c** | CATGGATTGCACATTTGAAGAC  GCAGGAGAAGAGAAGCTCTCA | Forward  Reverse |
| **PPAR-** α | TGTCACACAATGCAATCCGTT  ACAGTGTGTTCGTTGGCAA | Forward  Reverse |
| **TNF- α** | GATCTCAAAGACAACCAAATGTG  CTCAGCTGGAAGACTCCTCCCAG | Forward  Reverse |
| **IL-32** | TCAAAGAGGGCTACCTGGAGAC  TCTGTTGCCTCGGCACCGTAAT | Forward  Reverse |
| **Occluidn** | CTCGAGAAACTGAGTGCCTGGAC  AAGCTTTCGACCAATTCACCTGA | Forward  Reverse |
| **ZO-1** | ACTATGGCACATCAGCACG  TGGGCAAACAGACCAAGC | Forward  Reverse |
| **18S** | CATTCGAAGTCTGCCCTAT  GTTTCTCAGCTCCCTCTCC | Forward  Reverse |

**Supplementary Table 3**. Ingredient/ nutrient composition of Wistar rat methionine- and choline-deficient diet with high-fat diet diets (MCDHFD) (g/kg).

| **S.No** | **Ingredients Vitamin** | **Gm** |
| --- | --- | --- |
| 1 | Lard/ Saturated Fat | 330 |
| 2 | Casein | 256 |
| 3 | Sucrose | 55 |
| 4 | Choline Bitartrate | 0 |
| 5 | Maltodextrin | 60 |
| 6 | Corn Starch | 160 |
| 7 | Soybean Oil | 20 |
| 8 | L-Cysteine | 3.6 |
| 9 | Vitamin Mix | 35 |
| 10 | Cellulose | 60 |
| 11 | Potassium Citrate | 16.5 |
| 12 | Dicalcium Phosphate | 13 |
| 13 | Mineral Mix | 10 |
| 14 | Calcium Carbonate | 1.8 |

**Supplementary Table 4**. Vitamin composition of Wistar rat diets (g/kg).

| **S.No** | **Ingredients Vitamin** | **Gm** |
| --- | --- | --- |
| 1 | L- Methionine | 0.1 |
| 2 | L- Tryptophan | 1.8 |
| 3 | L- Histidine | 3.4 |
| 4 | L- Cystine | 3.7 |
| 5 | L- Threonine | 4.6 |
| 6 | L- Alanine | 5.1 |
| 7 | L- Tyrosine | 5.7 |
| 8 | L- Isoleucine | 6.1 |
| 9 | Glycine | 6.2 |
| 10 | L- Valine | 6.3 |
| 11 | L- Serine | 7.2 |
| 12 | L- Phenylalanine | 7.3 |
| 13 | L- Proline | 7.6 |
| 14 | L- Lysine HCL | 9.1 |
| 15 | L- Leucine | 10.5 |
| 16 | L- Aspartic Acid | 15.8 |
| 17 | L- Glutamic Acid | 28.9 |
